# Mucus plugs in chronic obstructive pulmonary disease are heterogeneous in size and shape and occur in relatively large airways

**DOI:** 10.1093/ajrccm/aamaf138

**Published:** 2026-01-23

**Authors:** Omar N Farooqui, Brett M Elicker, Travis S Henry, Lewis D Hahn, Kimberly G Kallianos, Meilan K Han, R Graham Barr, Benjamin M Smith, Alejandro P Comellas, Jessica Bon, Nadia Hansel, J Michael Wells, Robert Paine, Igor Barjaktarevic, Joyce D Schroeder, Prescott G Woodruff, Brendan K Huang, John V Fahy

**Affiliations:** Division of Pulmonary and Critical Care Medicine, Department of Medicine and Cardiovascular Research Institute, University of California, San Francisco, San Francisco, CA, United States; Division of Cardiac and Pulmonary Imaging, Department of Radiology and Biomedical Imaging, University of California, San Francisco, San Francisco, CA, United States; Division of Cardiothoracic Imaging, Duke Radiology, Duke University, Durham, NC, United States; Department of Radiology, Mayo Clinic, Rochester, MN, United States; Division of Cardiac and Pulmonary Imaging, Department of Radiology and Biomedical Imaging, University of California, San Francisco, San Francisco, CA, United States; Division of Pulmonary and Critical Care Medicine, Department of Medicine, University of Michigan, Ann Arbor, MI, United States; Department of Medicine, Columbia University Medical Center, New York, NY, United States; Department of Medicine, Columbia University Medical Center, New York, NY, United States; Division of Pulmonary, Critical Care, and Occupational Medicine, Department of Internal Medicine, University of Iowa, Iowa City, IA, United States; Section of Pulmonary, Critical Care, Allergy, and Immunologic Diseases, Department of Internal Medicine, Wake Forest University, Winston-Salem, NC, United States; Division of Pulmonary and Critical Care Medicine, Department of Medicine, Johns Hopkins School of Medicine, Baltimore, MD, United States; Division of Pulmonary, Allergy, and Critical Care Medicine, Department of Medicine, University of Alabama at Birmingham, Birmingham, AL, United States; Division of Respiratory, Critical Care and Occupational Pulmonary Medicine, Department of Medicine, University Utah School of Medicine, Salt Lake City, UT, United States; Division of Pulmonary and Critical Care Medicine, Department of Medicine, University of California, Los Angeles, Los Angeles, CA, United States; Department of Radiology, Mayo Clinic, Rochester, MN, United States; Division of Pulmonary and Critical Care Medicine, Department of Medicine and Cardiovascular Research Institute, University of California, San Francisco, San Francisco, CA, United States; Division of Pulmonary and Critical Care Medicine, Department of Medicine and Cardiovascular Research Institute, University of California, San Francisco, San Francisco, CA, United States; Division of Pulmonary and Critical Care Medicine, Department of Medicine and Cardiovascular Research Institute, University of California, San Francisco, San Francisco, CA, United States

**Keywords:** COPD, mucus plugs, emphysema

## Abstract

**Rationale:**

Mucus plugs occur in chronic obstructive pulmonary disease (COPD), but little is known about their size and airway location or whether these mucus plug features change as emphysema worsens.

**Methods:**

Computed tomographic lung scans from 31 participants with COPD from the SubPopulations and InteRmediate Outcomes In COPD Study (SPIROMICS) cohort were analyzed to quantify mucus plug size and airway location and extent of emphysema. Radiologist annotations of mucus plugs were incorporated in an image processing pipeline to generate size and location information. Emphysema was quantified as the percentage of voxels less than -950 Hounsfield units.

**Measurements and Main Results:**

The length distribution of 563 annotated mucus plugs varied from 2 to 50 mm. The distribution was multimodal, and an 11-mm length defined short (“stubby,” ≤11 mm) and long (“stringy,” >11 mm) plug phenotypes, with stubby plugs being most common (64%). Mucus plugs localized predominantly to airway generations 6 to 9, and their prevalence was highest in lower lobes. On a lobar basis, the mucus plug number decreased as emphysema percentage increased, whereas average mucus plug volume tended to increase.

**Conclusion:**

COPD is characterized by mucus plugs of varying size and shape, and emphysematous lung regions may be differentially susceptible to formation of these plugs. The location of mucus plugs in segmental and subsegmental airways in COPD makes them amenable to treatment with inhaled medications or bronchoscopy.

At a Glance Commentary **Scientific Knowledge on the Subject:**

Mucus plugs are common in COPD, but details of their number, size, and location features are unknown, as is the influence of emphysema on these features.

**What This Study Adds to the Field:**

This study shows that COPD is characterized by mucus plugs of varying size and shape and that emphysema has an influence on these features. Mucus plugs locate in segmental and subsegmental airways in COPD, making them amenable to treatment with inhaled therapeutics.

## Introduction

Mucus plug burden in computed tomography (CT) lung scans can be quantified in patients with airway disease using a scoring system based on bronchopulmonary segment anatomy.^1^ Mucus plug segment scores are high in patients with chronic obstructive pulmonary disease (COPD)^2^^,^^3^ and associate strongly with measures of airflow and multiple outcomes related to disease morbidity.^3^^,^^4^ High mucus plug scores even associate with all-cause mortality and lung-specific mortality in COPD.^5^ Together, these studies have drawn attention to the strong possibility that airway mucus plugs are causally related to mechanisms of airway obstruction and exacerbation, and they have heightened interest in COPD treatment strategies that target mucus plugs.^6^^,^^7^

Some unexpected findings have emerged from imaging studies that have highlighted mucus plug pathology in COPD. First, mucus plugs occur in patients who take inhaled bronchodilators and corticosteroids,^3^ indicating that the plugs are refractory to these treatments. Second, symptoms of cough and sputum production are not sensitive or specific indicators of radiographically identified mucus plugs.^3^ Third, mucus plugs associate with measures of airflow obstruction independent of severity of emphysema.^3^ Notably, patients with COPD and severe airflow obstruction can have high mucus plug scores and very limited emphysema,^3^ raising the possibility that mucus plugs are the dominant pathophysiologic mechanism of severe airflow obstruction in mucus plug– high/emphysema-limited patients.

Together, the recently published imaging data provide strong rationale for targeting mucus plugs with mucoactive treatment as a strategy to improve airflow and lung health in patients with COPD. Developing such treatment will be aided by more detailed information about the size and location features of mucus plugs in COPD.

Characterizing the size and shape of mucus plugs requires volumetric information. As recently described, the 3D geometry of airway mucus plugs and their location in the airways can be reconstructed from sequences of 2D CT lung images and methods of airway segmentation.^8^ We set out here to determine the size, shape, and airway location features of mucus plugs in COPD using these recently described methods. Because emphysema can alter airway caliber and distensibility,^9^ which could in turn change susceptibility to mucus plugging, we also set out to explore if emphysema percentage modifies the size features of mucus plugs. Some of the results of these studies have been previously reported in the form of an abstract.^10^

## Methods

### SPIROMICS

Participant data were obtained from the SPIROMICS (SubPopulations and InteRmediate Outcome Measures In COPD Study), a multicenter observational study designed to advance the understanding of COPD. The SPIROMICS protocol included detailed measures of lung function (including spirometry) and collection of multidetector CT lung scans (acquired after use of a bronchodilator), as previously described.^11^^,^^12^ A previous study reported on mucus plug burden data for a subset of CT lung scans in SPIROMICS.^3^ For the analyses presented here, 50 of these previously analyzed CT lung scans were selected. Scans were chosen randomly from the repository if they had a prior mucus plug score indicating mucus plugging. Following exclusion of scans with evidence of active infections, allergic bronchopulmonary aspergillosis, lung scarring, or motion degradation, 31 were further analyzed here (Figure S1).

### Emphysema percentage

The CT lung assessment system used by SPIROMICS has been described previously,^11^ as have methods for measuring emphysema and airway wall thickness.

Emphysema percentage was calculated at the lobar level as the percentage of lung voxels below -950 HU. To account for normal variation in emphysema percentage, derived reference equations were applied that included terms for age; sex; race and ethnicity; height; and body mass index category.^13^ For the present analysis, lobar-level emphysema measures were compared to corresponding mucus plug characteristics to evaluate associations between emphysema burden and mucus plugging.

### Mucus plug annotations

**Figure 1:**
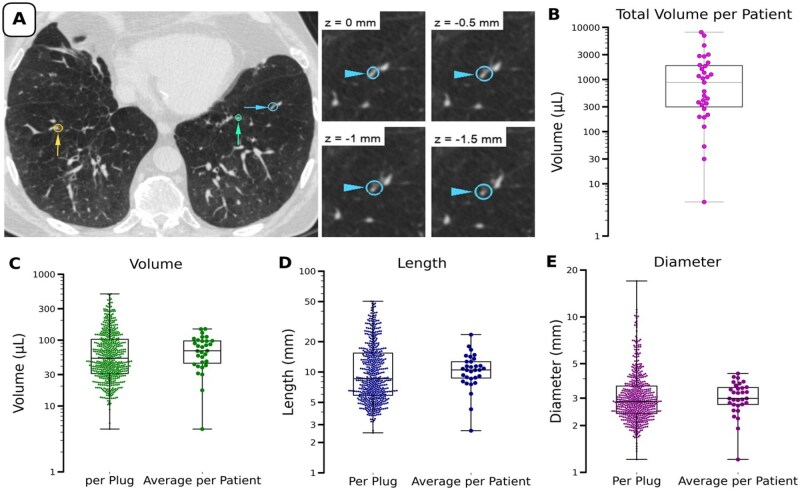
Mucus plugs are heterogeneous in size and shape in chronic obstructive pulmonary disease. (A) An ellipse placed over each plug generates a center coordinate, width, and height for a region of interest (colored arrows). The process is repeated at each axial slice, with z indicating the slice location relative to the initial image. (B) Total mucus volume per patient ranging from <1 mL to nearly 8 mL. (C-E) Results of shape feature quantification of individual mucus plugs (left, *n* = 563 plugs) and averages by patient (right, *n* = 31 patients), including plug volume (C), plug length (D), and plug diameter (E). Bars indicate the interquartile range, and whiskers show minimum and maximum values on a logarithmic scale.

Thoracic radiologists annotated mucus plugs in axial slices of CT lung scans, and the annotation process is illustrated in Figure 1A. The radiologists used a DICOM viewer (OsiriX; Pixmeo) to place a 2D elliptical region of interest over each mucus plug within an axial slice. Standardized visualization settings were employed with a window width of 1200 Hounsfield units (HU) and a window center of 600 HU. Axial voxel dimensions ranged from approximately 0.53 to 0.77 mm, with slice thickness varying between 0.63 and 0.75 mm and interslice spacing consistently measured at 0.5 mm. Each annotation included the center coordinate, width, and height of the region of interest, with plugs spanning multiple slices assigned a single numerical label to maintain consistency. Annotations for each scan were performed independently by 2 radiologists. Discrepancies, defined as plugs identified by only one radiologist, were resolved through adjudication by a third radiologist.

### Mucus plug segmentation, quantification, and visualization

To segment and analyze mucus plugs, the computational workflow previously published was utilized.^8^ In brief, each elliptical annotation was used to define a 2D region of interest around a mucus plug on a specific axial slice. Voxels from all annotated slices corresponding to a single plug were combined to form a volumetric representation. Segmentation of the mucus plug from surrounding lung parenchyma and airway lumen was performed using the Gustafson–Kessel fuzzy clustering algorithm, like that described for segmentation of lung nodules.^14^ Once segmented, the plugs were quantitatively analyzed for size and shape using both voxel-based and mesh-based techniques, as previously described.^15^ For visualization, the plug surface was reconstructed using the marching cubes algorithm, with surface smoothing applied prior to rendering.

### Lung and airway segmentation and skeletonization

Lung parenchyma was segmented on a lobar basis, as previously described,^8^ and airway segmentation was accomplished by a region-growing algorithm to estimate the central airways and a convolutional neural network method to enhance segmentation of smaller airways.^8^ Following segmentation, the airway tree was skeletonized to generate a centerline representation, which included segment level information for branch locations, segment lengths, local airway radii, generation numbers, and lobar assignments. As previously described, the airway termination points were identified as the most distal centerline points that did not have any further branching.^8^ To map each plug to a specific location within the airway tree, a Euclidean distance search was done to identify the nearest airway termination point for each plug, and the plugs were then integrated into the airway tree’s topological model. The airway generation of each plug was determined by counting the number of ­bifurcations from the trachea, with the trachea designated as generation zero.

### Statistical analyses

Statistical analyses were conducted using the SciPy, Statsmodels, and Scikit-learn packages in Python. Nonparametric numeric variables were assessed using Kruskal–Wallis, Mann–Whitney *U*, and Wilcoxon signed-rank tests. Categorical variables were evaluated using χ^2^ analysis. The association between numeric variables was quantified using the Pearson correlation coefficient (*r*). Linear regression was performed using ordinary least squares. Gaussian mixture models were used to assess multimodality in mucus plug length distributions, and the Akaike information criterion (AIC) was applied to identify the optimal number of components. Statistical significance was determined at *P* <.05.

## Results

### Mucus plugs in COPD are heterogeneous in length, diameter, and volume

The radiologists annotated 563 individual mucus plugs in CT scans from 31 participants with COPD, whose clinical details are summarized in Table 1. Mucus plugs were defined as complete occlusion of a bronchus, irrespective of generation or size. When parallel to the scan plane, mucus plugs were recognized as tubular densities with or without branching. When oriented obliquely or perpendicularly to the scan plane, they were identified as oval or rounded opacities seen on sequential slices and differentiated from blood vessels by their continuity with patent portions of the bronchial lumen and their position relative to adjacent blood vessels. To quantify the shapes and sizes of these mucus plugs, the voxels for each mucus plug were extracted, and the size of each plug was computed and quantified. The number of mucus plugs identified in the lungs of each of the 31 study participants is shown in Figure S2. We found that the length and diameter of these plugs varied greatly. The total volume of mucus plugs per patient ranged from <1 mL to nearly 8 mL (Figure 1B). Similarly, the volume of individual mucus plugs varied widely from approximately 4 to 503 µL (Figure 1C). The length of the mucus plugs ranged from about 2 to 50 mm (Figure 1D), while their diameter ranged from approximately 1 to 17 mm (Figure 1E). The average mucus plug length in a patient was inversely correlated with their smoking pack-year history (*r* = 0.33, *P* = .053) and their prebronchodilator FEV_1_ (*r* = −0.54, *P* = .001). The average plug length in the COPD patients with and without a history of asthma was similar (10.5 vs 11.9 mm, *P* = .6).

**Table 1:** Clinical characteristics of the COPD patients.

Characteristic	*N* = 31
**Age, y**	65 (50-77)
**Male sex**	15 (48.4%)
**Smoking pack-years**	50 (28-111)
** Current smoking**	10 (32.3%)
**GOLD stage**	
** 1**	1 (0.03%)
** 2**	2 (6.4%)
** 3**	18 (58.0%)
** 4**	10 (32.2%)
**CAT score**	18 (1-39)
**Current asthma**	8 (25.8%)
**Pre-BD FEV_1_ (%pred)**	28.9 (15.5-87.6)
**Pre-BD FVC (%pred)**	63.6 (45.1-112.9)
**Pre-BD FEV_1_/FVC**	0.33 (0.17-0.58)
**Post-BD FEV_1_ (%pred)**	32.9 (15.1-108.1)
**Post-BD FVC (%pred)**	73.6 (43.2-123.5)
**Post-BD FEV_1_/FVC**	0.35 (0.19-0.66)
**Airway wall thickness, mm**	1.1 (0.8-1.3)
**Race/ethnicity**	
** White**	27 (87.1%)
** Black/African American**	3 (9.7%)
** Asian**	1 (3.2%)
** Hispanic**	1 (3.2%)

Data are presented as No. (%) or median (range).

Abbreviations: %pred, percent predicted; BD, bronchodilator; CAT, COPD Assessment Test; COPD, chronic obstructive pulmonary disease; GOLD, Global Initiative for Chronic Obstructive Lung Disease; SPIROMICS, SubPopulations and InteRmediate Outcomes in COPD Study.

**Figure 2:**
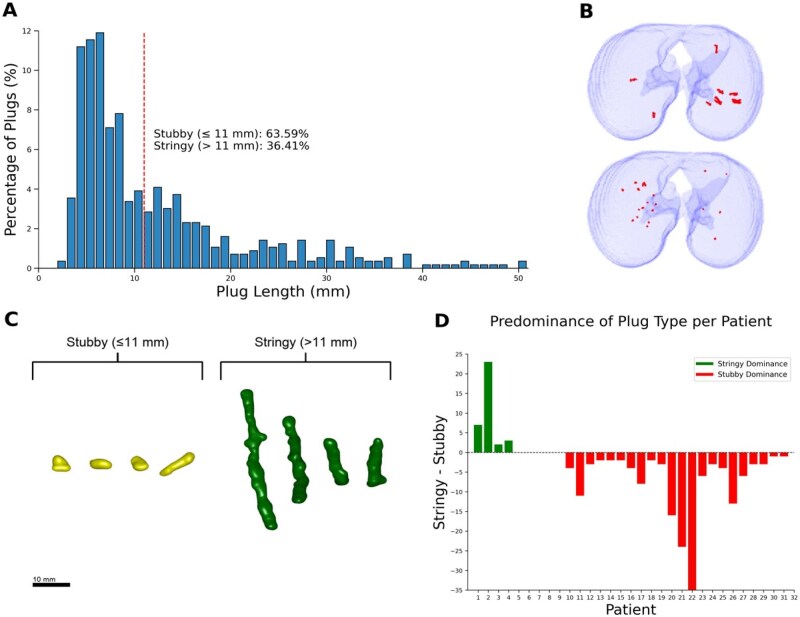
Mucus plugs have a bimodal length distribution. (A) The length distribution of mucus plug is multimodal. Short “stubby” plugs predominate, whereas longer (“stringy”) mucus plugs are less common. (B) Axial projection of 3D lung volume highlighting the X-Y spatial distribution of plug phenotypes in computed tomography (CT) lung scans from 2 different patients. The upper rendering image is from the CT lung scan of a patient with a predominance of stringy plugs and the lower rendering image is from a patient with a predominance of stubby plugs. (C) Representative 3D reconstructions of mucus plugs categorized as stubby (yellow) and stringy (green), illustrating the morphological differences between the 2 types. (D) Per-patient data for mucus plug phenotypes presented as the per-patient difference in the number of stringy and stubby plugs. Positive values on the y-axis occur when stringy plugs predominate within a patient, whereas negative values occur when stubby mucus plugs predominate. The graph overall shows that most of the 31 chronic obstructive pulmonary disease patients had a predominance of stubby mucus plugs.

We noted that the distribution of mucus plug lengths was multimodal (Figure 2A), and assessment of model fit by AIC revealed that a Gaussian mixture model with 3 underlying distributions had the highest likelihood. Based on a length of 11 mm separating the 2 dominant populations in the model, we divided the plugs into short plugs that were ≤11 mm in length and long plugs that were >11 mm in length. Using previously established labels for mucus plugs in patients with asthma,^5^ we classified longer mucus plugs as “stringy” and shorter ones as “stubby.” Among the 563 plugs analyzed, 358 (63.6%) were classified as stubby, while 205 (36.4%) were stringy (Figure 2A). Rendering all of the mucus plugs from a patient with a predominance of stubby plugs and another with a predominance of stringy plugs illustrates how the plugs distribute in the X-Y plane of the lungs and how select phenotypes appear in 3 dimensions (Figure 2B, C). The predominance of plug type per patient is shown in Figure 2D, highlighting whether individual patients had more stringy or stubby mucus plugs, with most patients having a predominance of stubby plugs.

### Mucus plugs in CT lung images primarily localize to airways that are 2 to 4 mm in diameter

**Figure 3:**
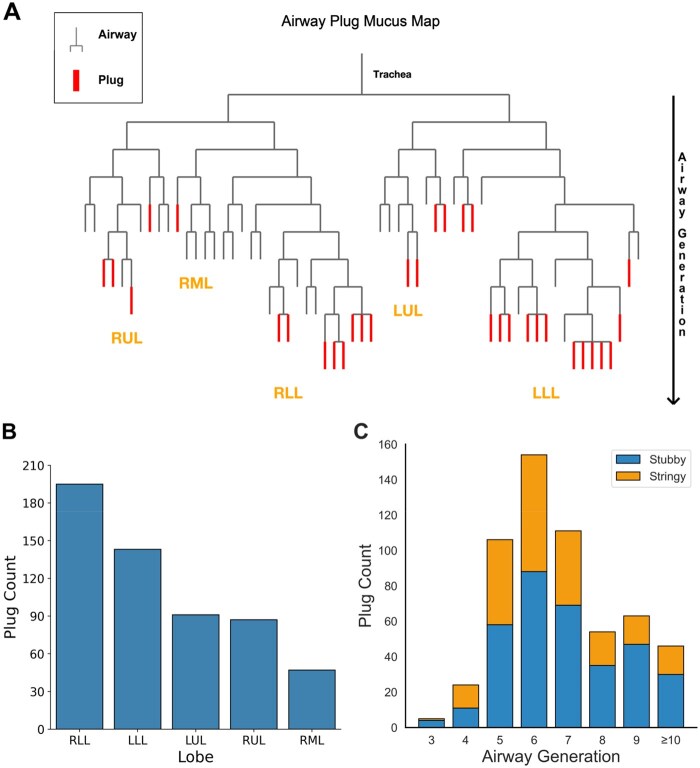
Mucus plugs are primarily located in proximal airway generation in chronic obstructive pulmonary disease. (A) Specific airway location information for each mucus plug allows generation of an “airway mucus plug map.” This map is a schematic representation of the airway tree that shows the location of each mucus plug (red) in airways of the right upper lobe (RUL), right middle lobe (RML), right lower lobe (RLL), left upper lobe (LUL), and left lower lobe (LLL). (B) The number of airway mucus plugs varies in different lung lobes. Mucus plug numbers are highest in the RLL. (C) Mucus plugs are located primarily in airway generations 6 to 9, which have a diameter of 2-4 mm. Stubby plugs (≤11 mm in length) predominate in all generations except generation 4.

By segmenting lung parenchyma and airways on a lobar basis, every mucus plug could be localized to a specific airway branch and lobe. This information allowed generation of an “airway mucus plug map” for each patient in which the location of each mucus plug within the branching airway tree is shown (Figure 3A). In this way we found that the number of mucus plugs in the lower lobes was greater than in the upper and middle lobes (Figure 3B). Furthermore, a frequency distribution plot summarizing the airway generations occluded by all 563 mucus plugs revealed that they primarily locate in airway generations 4 to 9 (Figure 3C).

### Mucus plug size and emphysema percentage

**Figure 4:**
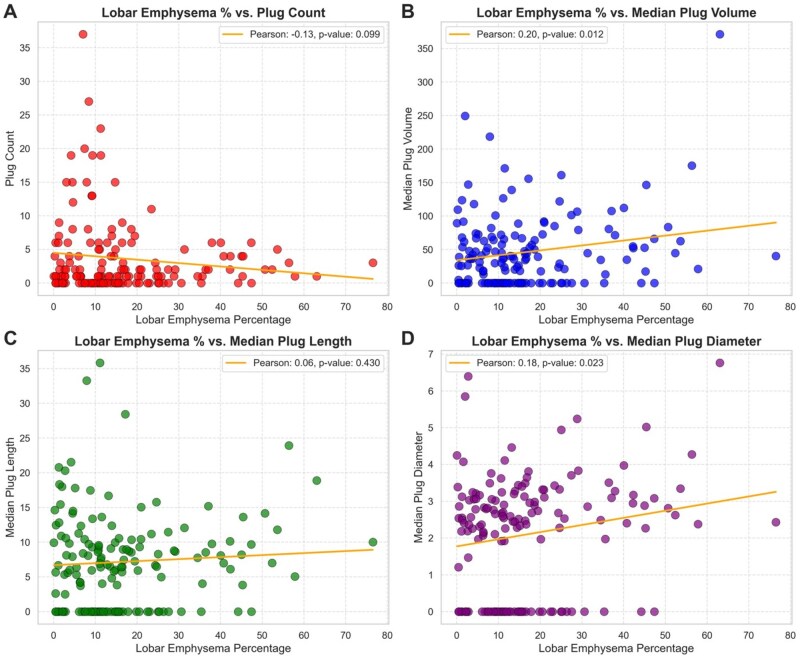
The size and location features of mucus plugs in chronic obstructive pulmonary disease are influenced by emphysema. (A) The number of mucus plugs tends to decrease in lung lobes as the emphysema percentage increases. (B) The median volume of individual mucus plugs tends to increase as the emphysema percentage in a lung lobe increases. (C) The median length of individual mucus plugs does not vary significantly as the emphysema percentage in a lung lobe increases. (D) The median diameter of mucus plugs shows a positive relationship with emphysema percentage.

To explore the relationships between emphysema and mucus plug number, size, and location, we used lobar-level data. We found that as emphysema percentage increased, the mucus plug number tended to decrease whereas the volume of mucus plugs increased (Figure 4A, B). This emphysema-associated increase in mucus plug volume was driven by an increase in plug diameter because emphysema percentage did not significantly influence the length of mucus plugs (Figure 4C, D).

## Discussion

Previous studies of mucus plugs in COPD have used the mucus plug segment score to quantify mucus plug burden.^3^ Although a highly useful measure of the total burden of mucus plugs in the lung, the segment score does not provide size, shape, or airway tree location information. Such information is required to inform the development of needed mucoactive treatments for COPD. Here we provide detailed size and shape information on 563 mucus plugs in 31 patients with COPD and we identified the airway tree locations occluded by these plugs. We show that radiographically visible mucus plugs in COPD are highly heterogeneous in size and shape and that they locate in subsegmental airways that are consequential for airflow. We also provide suggestive evidence that the number and size of mucus plugs in COPD is influenced by the severity of emphysema.

We found that the length distribution of mucus plugs in COPD is bimodal, and best-fit modeling showed that a plug length of 11 mm defines short (“stubby”) and long (“stringy”) plug phenotypes. Most of the mucus plugs were stubby and most patients had more stubby plugs than stringy plugs. It is logical to assume that stubby mucus plugs will have quicker responses to mucoactive treatments and that stringy plugs, some of which have lengths exceeding 40 mm, will take longer to treat or may be recalcitrant to treatment. For this reason, it would be helpful to have size information on mucus plugs in clinical trials of mucoactive drugs to determine if length features of plugs predicts the time kinetics of treatment response. Similarly, the airway tree location information that we provide here is relevant for informed decision making about optimal treatments. We show that mucus plugs range in their location from second to ninth order bronchi and that there is a higher prevalence of plugs in the lower lobes. These location features have potential to influence treatment responses to mucoactive drugs. For example, the higher prevalence of plugs in the lower lobes may make them more susceptible to inhaled treatments because inhaled aerosols tend to deposit more in lower lobes than in upper lobes.^16^ Also relevant for inhaled treatments is the airway generation of the plugs. More distal plugs may be more resistant to treatment with inhaled drugs because drug deposition decreases with airway generation. Together, the size and location data for mucus plugs that we present here illustrate the utility of detailed mucus plug phenotyping in COPD. These data provide a rationale for quantifying mucus plug size metrics in clinical trials of mucoactive drugs for COPD, in order to better understand how mucus plug phenotypes influence treatment responses.

The pathology of COPD includes mucus plugs and emphysema. We found that regional emphysema severity (lobe-level) was associated with fewer mucus plugs, even as the average volume of individual mucus plugs was higher in these lobes. While the present study did not investigate mechanisms or temporality, our observations suggest that emphysematous lung regions may be differentially susceptible to mucus plug formation/subtype or vice versa. Clinical trials of mucoactive treatments should shed light on the interplay of mucus plug and emphysema pathobiology in COPD.

The data for the size of mucus plugs in COPD patients that we report here are similar to the size data that we have reported previously for patients with asthma.^8^ First, our finding that COPD mucus plugs locate primarily to airway generations 6 to 9 mirrors the findings for asthma mucus plugs.^8^ Second, the length and diameter of the plugs in both diseases are similar and the range of values for total mucus plugs volume is also similar.^8^ Notably, this total mucus plug volume range (approximately 0.5-5.0 mL) is relatively low compared to the total lung volume of mucus that occurs in cystic fibrosis (CF) and non-CF bronchiectasis where relatively large amounts of mobile mucus within larger airways occurs in conjunction with more static mucus plugs in subsegmental airways.^17^ Finally, the pathology of chronic mucus plugs in both asthma and COPD is similar and characterized by a mixture of MUC5AC and MUC5B mucins, infiltration with granulocytes, and presence of extracellular DNA traps.^18^

The size and location information that we provide here for mucus plugs in COPD is relevant to decisions about dosing and delivery of mucoactive drugs and the selection of drug candidates. The reason the information is important is that drug development decisions related to the dose and formulation characteristics of drugs, especially inhaled drugs, requires knowledge of the volume of mucus being targeted and the location of the mucus in order to calculate drug dose and drug characteristics such as particle size. In addition, the quantification of mucus plug size pre- and posttreatment in early-stage trials of novel drugs can provide important information about the duration of time needed to lyse mucus plugs.

A limitation of our study is that it only characterized mucus plugs in 31 patients with COPD. The number of scans needed to be limited because of the labor-intensive nature of plug annotations in each of the axial slices of these scans (average of 640 axial slices per scan). However, these 31 patients provided a total of 563 mucus plugs for characterization, which provides confidence that the data generated is robust.

In summary, we report that airway mucus plugs in COPD are heterogeneous in size and shape, occur in relatively large airways, and are more prevalent in the lower lobes. In addition, more severe emphysema is associated with fewer but larger mucus plugs. These data provide rationale for measuring the size and location features of mucus plugs as predictive and monitoring biomarkers in clinical trials of needed mucoactive drugs in COPD to guide optimal dosing regimens for these treatments.

## Supplementary Material

aamaf138_Supplementary_Data

## Data Availability

This article has an online data supplement, which is accessible at the Supplements tab.
