# Supplementary material for: Mucus plugs in chronic obstructive pulmonary disease are heterogeneous in size and shape and occur in relatively large airways
Source: Am J Respir Crit Care Med. 2026 Jan 23;212(2):268–76. doi: 10.1093/ajrccm/aamaf138 (PMC13051690; doi:10.1093/ajrccm/aamaf138)
Supplement: Supplementary file 1 [file aamaf138_supplementary_data.pdf]

ONLINE SUPPLEMENT

MUCUS PLUGS IN COPD ARE HETEROGENEOUS IN SIZE AND SHAPE AND OCCUR IN RELATIVELY LARGE AIRWAYS

Omar N. Farooqui[1], Brett M. Elicker[2], Travis S. Henry[3], Lewis D. Hahn[4], Kimberly G. Kallianos[2], MeiLan K. Han[5], R. Graham Barr[6], Benjamin M. Smith[6], Alejandro P. Comellas[7], Jessica Bon[8], Nadia Hansel[9], J. Michael Wells[10], Robert Paine III[11], Igor Barjaktarevic[12], Joyce D. Schroeder[13], Prescott G. Woodruff[1], Brendan K. Huang[1], John V. Fahy[1]

INSTITUTIONAL AFFILIATIONS

[1]Division of Pulmonary and Critical Care Medicine, Department of Medicine and Cardiovascular Research Institute, University of California San Francisco, San Francisco, California, USA.

[2]Division of Cardiac and Pulmonary Imaging, Department of Radiology and Biomedical Imaging, University of California San Francisco, San Francisco, California, USA.

[3]Division of Cardiothoracic Imaging, Duke Radiology, Duke University, Durham North Carolina, USA.

[4] Department of Radiology, University of California San Diego, La Jolla, California, USA. [5]Division of Pulmonary and Critical Care Medicine, Department of Medicine, University of Michigan at Ann Arbor, Ann Arbor, Michigan, USA.

[6]Department of Medicine, Columbia University Medical Center, New York, New York, USA.

[7]Division of Pulmonary, Critical Care, and Occupational Medicine, Department of Internal Medicine, University of Iowa, Iowa City, Iowa, USA.

[8]Section of Pulmonary, Critical Care, Allergy, and Immunologic Diseases, Department of Internal Medicine, Wake Forest University, Winston-Salem, North Carolina, USA.

[9]Division of Pulmonary and Critical Care Medicine, Department of Medicine, Johns Hopkins School of Medicine, Baltimore, Maryland, USA.

[10]Department of Pulmonary, Allergy, & Critical Care Medicine, Department of Medicine, University of Alabama at Birmingham, Birmingham, Alabama, USA.

[11]Division of Respiratory, Critical Care and Occupational Pulmonary Medicine, Department of Medicine, University Utah School of Medicine, Salt Lake City, Utah, USA.

[12]Division of Pulmonary and Critical Care Medicine, Department of Medicine, University of California Los Angeles, Los Angeles, California, USA.

[13]Department of Radiology, Mayo Clinic, Rochester, Minnesota, USA.

*Address Correspondence to: John Fahy, M.B., M.D., M.Sc., Room 1307, Health Sciences East, University of California, San Francisco, 513 Parnassus Ave, San Francisco, CA 94143.Tel: +1 415-476-9940; Fax: +1 415-476-5712; email: john.fahy@ucsf.edu

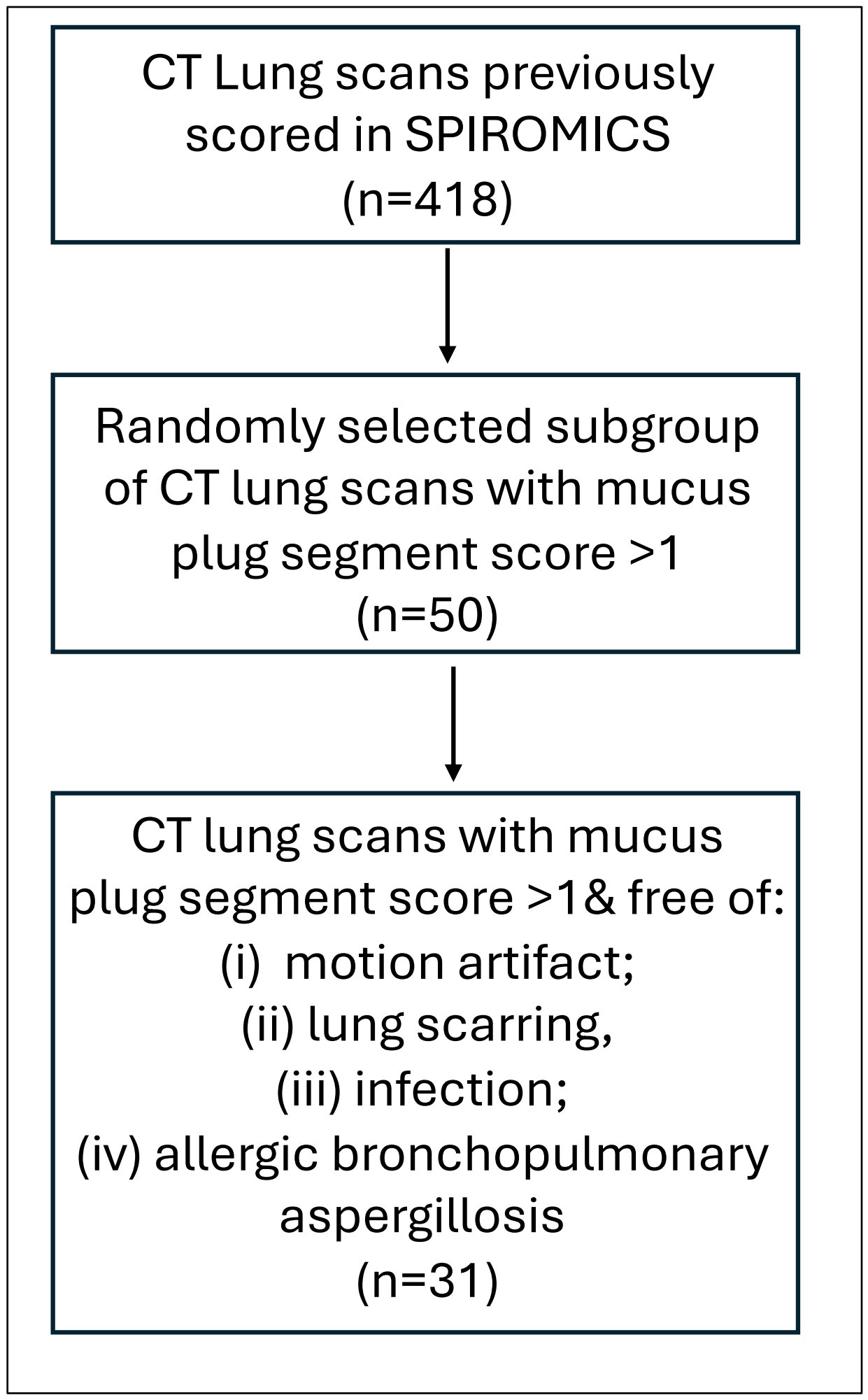

Figure S1: Selection of CT lung scans for inclusion in the study

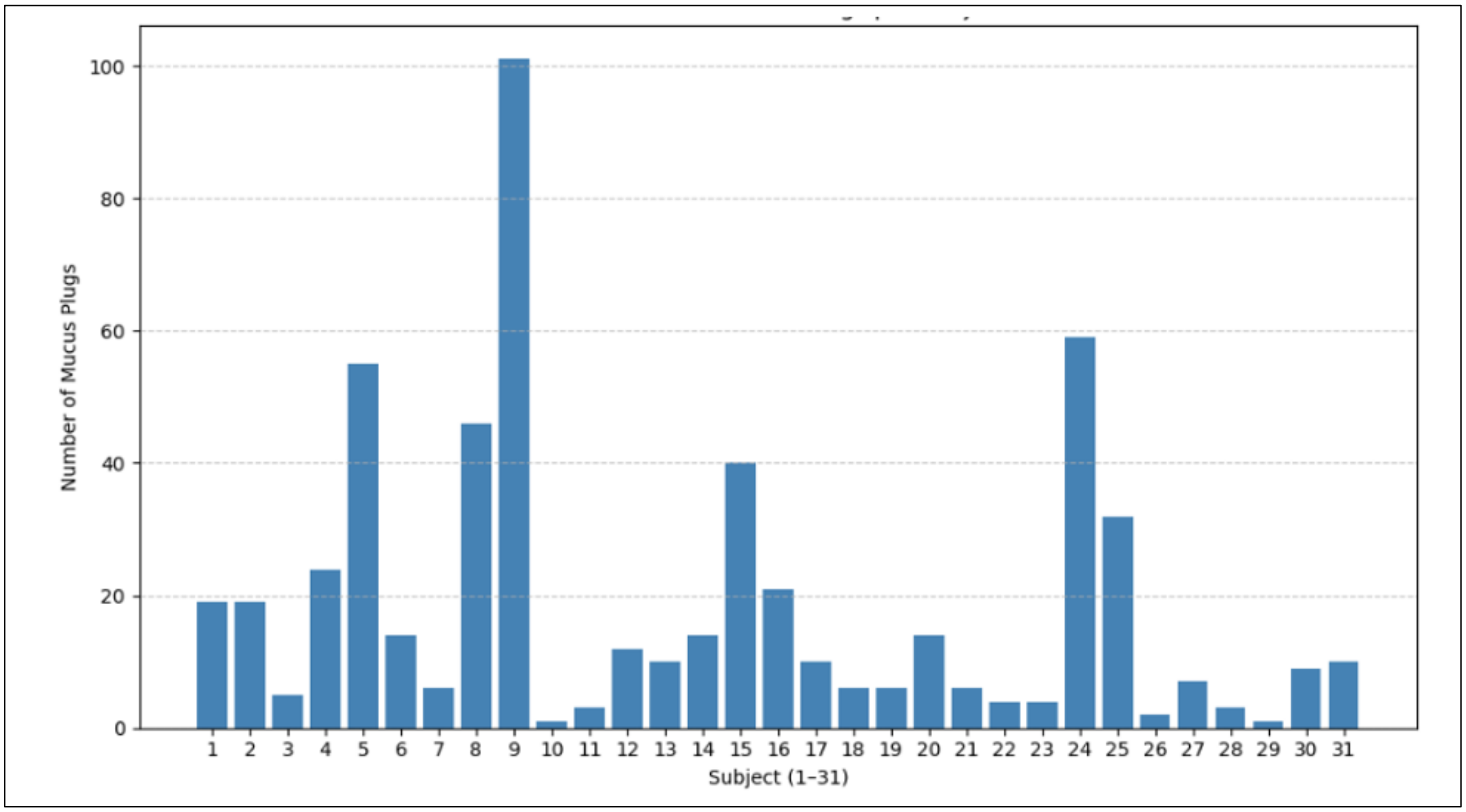

Figure S2: Frequency distribution plot showing the number of mucus plugs identified in the lungs of each of the 31 study participants
